# Evaluation of the accuracy of bacterial genome reconstruction with Oxford Nanopore R10.4.1 long-read-only sequencing

**DOI:** 10.1099/mgen.0.001246

**Published:** 2024-05-07

**Authors:** Nicholas D. Sanderson, Katie M.V. Hopkins, Matthew Colpus, Melody Parker, Samuel Lipworth, Derrick Crook, Nicole Stoesser

**Affiliations:** 1 NIHR Oxford Biomedical Research Centre, University of Oxford, Oxford, UK; 2 Nuffield Department of Medicine, University of Oxford, Oxford, UK; 3 NIHR Health Protection Research Unit in Healthcare Associated Infections and Antimicrobial Resistance at University of Oxford in partnership with Public Health England, Oxford, UK

**Keywords:** genome sequencing, hybrid assembly, long-read assembly

## Abstract

Whole-genome reconstruction of bacterial pathogens has become an important tool for tracking transmission and antimicrobial resistance gene spread, but highly accurate and complete assemblies have largely only historically been achievable using hybrid long- and short-read sequencing. We previously found the Oxford Nanopore Technologies (ONT) R10.4/kit12 flowcell/chemistry produced improved assemblies over the R9.4.1/kit10 combination, however long-read only assemblies contained more errors compared to Illumina-ONT hybrid assemblies. ONT have since released an R10.4.1/kit14 flowcell/chemistry upgrade and recommended the use of Bovine Serum Albumin (BSA) during library preparation, both of which reportedly increase accuracy and yield. They have also released updated basecallers trained using native bacterial DNA containing methylation sites intended to fix systematic basecalling errors, including common adenosine (A) to guanine (G) and cytosine (C) to thymine (T) substitutions. To evaluate these improvements, we successfully sequenced four bacterial reference strains, namely *Escherichia coli*, *Klebsiella pneumoniae*, *Pseudomonas aeruginosa* and *Staphylococcus aureus*, and nine genetically diverse *E. coli* bloodstream infection-associated isolates from different phylogroups and sequence types, both with and without BSA. These sequences were *de novo* assembled and compared against Illumina-corrected reference genomes. In this small evaluation of 13 isolates we found that nanopore long-read-only R10.4.1/kit 14 assemblies with updated basecallers trained using bacterial methylated DNA produce accurate assemblies with ≥40×depth, sufficient to be cost-effective compared with hybrid ONT/Illumina sequencing in our setting.

**Impact Statement**

Currently, the best method of building accurate and complete bacterial genome assemblies is to create a hybrid assembly, i.e. combining information from both long and short sequencing reads. Short-reads are highly accurate but can be difficult to assemble into a complete genome because they do not effectively span repetitive sequences. Long-reads have traditionally been less accurate, but help in determining genome structure. By combining long- and short-reads, we get high accuracy and complete structural information. However, this also involves multiple sequencing platforms and more resource than using a single-sequencing platform. In this study, we compare long-read-only assemblies using Oxford Nanopore Technology’s (ONT) updated chemistry and software to hybrid Illumina-Nanopore assemblies. We sequenced four bacterial pathogens with published reference genomes (*Staphylococcus aureus, Klebsiella pneumoniae, Pseudomonas aeruginosa*, and *Escherichia coli*) and 12 bloodstream infection-associated *E. coli* isolates, and show that assemblies from the latest technology are able to compete with hybrid Illumina-Nanopore assemblies in their quality, representing a significant step i n bacterial genome assembly using ONT data only.

## Data Summary

Assemblies have been made available at: https://doi.org/10.6084/m9.figshare.25472167.v1.Code available at: https://gitlab.com/ModernisingMedicalMicrobiology/assembly_comparison.

## Introduction

Generating high-quality whole-genome *de novo* assemblies for bacterial pathogens has become an important tool supporting diagnostic, infection prevention control and public health initiatives, improving our understanding of antimicrobial resistance and pathogen epidemiology [1]. The introduction of longer sequencing reads from PacBio and Oxford Nanopore Technologies (ONT) has enabled the reconstruction of previously difficult-to-assemble genomes. Previously we have found the most cost-effective method of assembling high-accuracy bacterial genomes is to combine long R9.4.1/kit10 ONT reads with high-accuracy short Illumina reads [2]. Generating these high-quality assemblies without the need for Illumina sequencing could reduce costs and laboratory time, and this would enable more cost-effective, faster, and higher-throughput sequencing.

ONT have iteratively updated their flowcell technology and chemistry from R9.4.1/kit10 through R10.3/kit12 to R10.4/kit12 and most recently R10.4.1/kit14; older flowcells and chemistries have been phased out. Further changes to ONT workflows have included an increase in sampling rate on the nanopore devices from 4 k samples per second to 5 kHz, which generates more data points for basecalling and thereby improves accuracy, according to ONT [3]. Users of R10.4 have reported cytosine to thymine (C-to-T) and guanine to adenine (G-to-A) errors, hypothesized to be caused by methylated sites confusing the basecalling models. ONT have released updated basecalling models trained with native bacterial DNA and designed to fix errors associated with methylated sites, which also takes advantage of the increased sampling rate. It is now also recommended to use bovine serum albumin (BSA), which is added during library preparation to improve sequencing yield and quality by blocking non-specific binding sites.

We have previously compared ONT-only assemblies using R10.3 or R10.4 sequencing data to the R9.4.1/Illumina hybrid assemblies [4]. We looked at four common, diverse human pathogenic reference strains representing a range of %GC content, namely, *Staphylococcus aureus* MRSA252*, Klebsiella pneumoniae* MGH78578*, Pseudomonas aeruginosa* PAO1, and *Escherichia coli* CFT073. R10.4-only assemblies (using superior accuracy [sup] basecalling) looked promising in terms of genome reconstruction, error rates and gene recovery, but generally required deeper long-read sequencing depths than for a hybrid assembly, rendering the latter more cost-effective and high-throughput if access to both ONT and Illumina sequencing modalities was available [4]. Smaller plasmids (<5 kb) were also less effectively recovered as part of long-read-only sequencing. These findings have been consistent with other more recent studies [5,6].

As an update to our previous evaluation, here we compare the accuracy of assemblies from nanopore-only R10.4.1 sequencing with our previous hybrid R9.4.1/Illumina assemblies for the same four species references. Given that *E. coli* was the most challenging species to assemble previously, we also evaluate the accuracy of R10.4.1-only assemblies for 12 *E. coli* strains representing major phylogroups of the species compared with R9.4.1/Illumina hybrids created for these *E. coli* strains. As part of this second evaluation, we characterize the impact of BSA on sequencing output, the impact of increased sampling rate and the newer basecalling models on read and assembly accuracy, and estimate current sequencing costs per isolate using R10.4.1 long-read-only assembly.

## Methods

### Bacterial isolates and DNA extraction

DNA was extracted from laboratory stocks of four bacterial reference strains: *P. aeruginosa* PAO1*, S. aureus* MRSA252*, K. pneumoniae* MGH78578*, E. coli* CFT073, and 12 bloodstream-associated *E. coli* isolates, for which we had existing Illumina data [7] and which were chosen to reflect the genetic diversity within the species based on *in silico* phylotyping [8]. Stocks were stored at −80 °C in nutrient broth with 10 % glycerol. Stocks were cultured on Columbia Blood Agar overnight at 37 °C. One colony was selected and sub-cultured for DNA extraction using the QIAGEN Genomic Tip 100 G^−1^ kit. Extracts were stored in elution buffer at 4 °C for the duration of the study. DNA quality was assessed with the Qubit Fluorometer and TapeStation immediately after extraction, and periodically prior to sequencing thereafter.

### Nanopore sequencing

All sequencing occurred on an ONT GridION device (Minknow version 23.04.5) using a 5 kHz sampling rate with R10.4.1 flow cells. Sequencing libraries were created using the Rapid Barcoding (RBK114.96) kit, and each run was set for 72 h.

For the four reference strains, we followed the recommendation to use BSA (50 mg ml^−1^, Thermo Fisher Scientific, AM2616) as part of library preparation and created a multiplexed library using the four reference strains, which was run on a single R10.4.1 flowcell. The yield for *K. pneumoniae* on this run was too low (10.8 Mb only), so this extract was sequenced again as a single sample on a second R10.4.1 flowcell. To specifically assess the impact of adding BSA to the flowcell priming mix, a multiplexed library of the 12 *E. coli* isolates was created, and split between two R10.4.1 flowcells. One of these flowcells included the addition of BSA to the flowcell priming mix, and the other did not. Sampling rates, basecalling and runtimes were as above.

R9.4.1 data were generated previously [4].

### Illumina sequencing

Illumina sequencing data for the reference strains were generated previously as part of [4], and for the 12 *E. coli* isolates as part of [7].

A summary of the experimental workflow is shown in Fig. S1, available in the online version of this article.

### Generating high-quality reference genomes for comparisons

#### Species reference strains

Historically deposited reference sequences may contain ambiguous nucleotide codes (e.g. R [A or G], Y [C or T], W [A or T] or K [G or T]; IUPAC) and long-term storage of frozen stocks of reference isolates may lead to genuine genetic changes, particularly in the tRNA and 23S/16S rRNA regions (Figs S2–S4). Therefore, for this study we generated reference genome sequences *de novo* for the reference strains of *E. coli* CFT073 (AE014075.1), *K. pneumoniae* MGH78578 (CP000647.1), *P. aeruginosa* PAO1 (NC_002516.2), *S. aureus* MRSA252 (NC_002952.2) by assembling Illumina sequence data from [8] and R9.4.1 nanopore data from [4] using Trycycler [9] (version 0.5.4) and following the default workflow, with assembly inputs from flye [10] (version 2.9.3-b1797), miniasm [11] (version 0.3-r179), and raven [12] (version 1.8.3). Contigs were ONT polished with medaka (version 1.6.0; default settings; https:// github.com/nanoporetech/medaka) and Illumina polished with polypolish [13] (version 0.6.0) and POLCA [14] (version 4.1.1). The R9.4.1 nanopore data was used to generate a reference sequence independently of the experimental R10.4.1 data being considered in this study. Notably however, Trycycler was unable to resolve the five plasmid sequences present in the *K. pneumoniae* reference and therefore Illumina-corrected (using sequence data from [8] and the SNIPPY workflow [version 4.6.0; https://github.com/tseemann/snippy]), versions of the original plasmid sequences (CP000648.1, CP000649.1, CP000650.1, CP000651.1, CP000652.1) were used in the comparison.

#### *E. coli* isolates

For the 12 clinical *E. coli* isolates where no gold standard reference genomes are publicly available, hybrid Illumina/nanopore assemblies were generated as references using R9.4.1/Illumina data with Unicycler (v0.5.0, default settings, except G_117, E_8318 where flye assemblies were used instead of miniasm within Unicycler) [15]. Trycycler was not used as it required too much manual intervention to resolve the genomes and the difference noted for three of nine cases where it was evaluated to be minimal (zero SNVs or INDELs for B1_2599, ten for E_831, and two for F_624 compared to R9.4.1/Illumina hybrids assembled with Unicycler). The Unicycler reference assemblies were compared to R10.4.1 long-read-only assemblies for the same isolates, generated using the optimal strategy developed on the four mixed species reference strains, namely Dorado v0.5.3 basecalling +Flye assembly with a single round of Medaka polishing.

### Sequence data processing and comparisons with reference sequences

An analysis workflow was written in nextflow and is publicly available in gitlab (https://gitlab.com/ModernisingMedicalMicrobiology/assembly_comparison).

#### Basecalling

Raw R9.4.1 nanopore data from [8] was re-basecalled using guppy version 5.0.12+eb1 a981 with the dna_r9.4.1_e8.1_sup.cfg model. R10.4.1 nanopore raw data was basecalled using either Dorado version 0.4.0+0aaf16d and dna_r10.4.1_e8.2_400bps_sup@v4.2.0 (Dorado_0.4.0) or Dorado version 0.5.3+d9af343 and dna_r10.4.1_e8.2_400bps_sup@v4.3.0 (Dorado_0.5.3) models, which included the methylation aware basecalling models (v4.3) released in Dorado version 0.5.0 (https://github.com/nanoporetech/dorado/releases/tag/v0.5.0).

#### Comparisons with reference sequences

Per read accuracy, subsampling, assembly, contig reference comparison and gene recovery metrics were all evaluated as described previously [4].

For empirical read accuracy, reads were mapped to the hybrid ONT-Illumina trycycler assembled reference sequences with minimap2 [16] (version 2.22-r1101) and samtools [17], and the percentage identity was calculated from the query distance (NM tag) in the bam file over the query length, multiplied by 100. Empirical Q-scores were not shown due to the number of reads with no errors resulting in infinite values that skewed the results.

For R10.4.1 long-read-only assemblies, nanopore reads for each species reference strain or clinical *E. coli* isolate were subsampled (20 to 100×depths at 10×intervals) using Rasusa (v0.6.1) [18], then assembled with Flye [10] (using the --meta and –nano-hq parameters, version 2.9-b1768) along with the un-subsampled reads (represented in figures as ‘0’). Medaka (1.6.0; default settings; https://github.com/nanoporetech/medaka) was used to polish the assembled contigs from Flye. Only one polishing iteration was used as further polishing cycles did not improve error rates and used significant compute resource (Fig. S5 [also shown in our previous evaluation [4]]).

To evaluate if hybrid assembly represented an advantage over R10.4.1 long-read-only assembly, the four species reference strains were hybrid assembled with nanopore and Illumina sequences using Unicycler (v0.5.0, default settings) [15]. Hybrid assemblies were generated with both R9.4.1 and R10.4.1 nanopore data for comparison. The nanopore reads were subsampled to specific depths but the complete quantity of Illumina reads was always used.

#### Assembly error profiling

The error types from the assembled contigs were characterized using the output from DNADIFF (Mummer3 package [19]) and summarized based on type insertion or deletion (INDEL) and single nucleotide differences (SNV).

### Statistical analysis and visualizations

Mann–Whitney–Wilcoxon (two-sample Wilcoxon) tests were used to evaluate the statistical significance of pairwise differences in continuous non-normal variables such as read length and accuracy for each sequencing modality. Statistical analysis and data visualizations were carried out in R version (version 4.3.0) using the ggpubr and ggplot packages, and Fig. S1. was generated in Biorender (www.biorender.com).

## Results

### Four bacterial species reference strains: sequencing yield and read length distributions

The R9.4.1 flowcell generated more data than the combined R10.4.1 flowcells after running for 72 h, (10.5 Gb versus 5.2 Gb, Fig. S6 and Table 1). The data generated for the two R10.4.1 results and plotted originate from the same combined read yields from two flowcells (as *K. pneumoniae* was re-sequenced separately) but differ by basecalling method (Dorado_0.4.0 versus Dorado_0.5.3), resulting in slightly different total base outputs (Fig. S6).

**Table 1.** Total bases, and median (IQR) read lengths excluding the unclassified barcode, per flowcell and per species isolate on each flowcell

Run type	Flowcell bases	Species	Q1 length	Median length	Q3 length	Isolate bases
r9.4.1 4 k BSA- Rapid Guppy SUP	10 538 807 369	*E. coli*	1 876.25	4 263.50	9 026.25	2 242 222 750
		*K. pneumoniae*	2 755.25	6 101.00	14 104.50	3 458 646 526
		*P. aeruginosa*	2 857.50	7 716.50	16 292.50	4 138 688 286
		*S. aureus*	3 849.50	11 132.00	23 431.75	699 249 807
r10.4.1 5 k BSA +Rapid Dorado_0.4.0 SUP	5 238 710 880	*E. coli*	416.25	1 914.00	8 113.75	2 617 431 144
		*K. pneumoniae*	489.75	2 048.50	7 186.00	417 791 092
		*P. aeruginosa*	250.75	473.00	3 332.00	1 660 043 947
		*S. aureus*	745.50	3 533.50	11 131.25	543 444 697
r10.4.1 5 k BSA +Rapid Dorado_0.5.3 SUP	5 118 562 131	*E. coli*	254.00	1 392.00	7 126.25	2 544 407 444
		*K. pneumoniae*	302.00	2 016.50	6 979.50	430 622 739
		*P. aeruginosa*	164.00	405.50	3 760.50	1 619 506 365
		*S. aureus*	536.50	3 308.00	10 561.75	524 025 583

Median read lengths varied between species for each run type with R9.4.1 having the highest median and IQR lengths across all species (median read length for R9.4.1 : 6.5 kb [IQR: 2.68–15.2 kb] versus for R10.4.1 : 1.7 kb [IQR: 0.34–7.73 kb]; *P*<0.001, two-sample Wilcoxon) (Fig. S7).

### Four bacterial species reference strains: empirical read accuracy

**Fig. 1.**
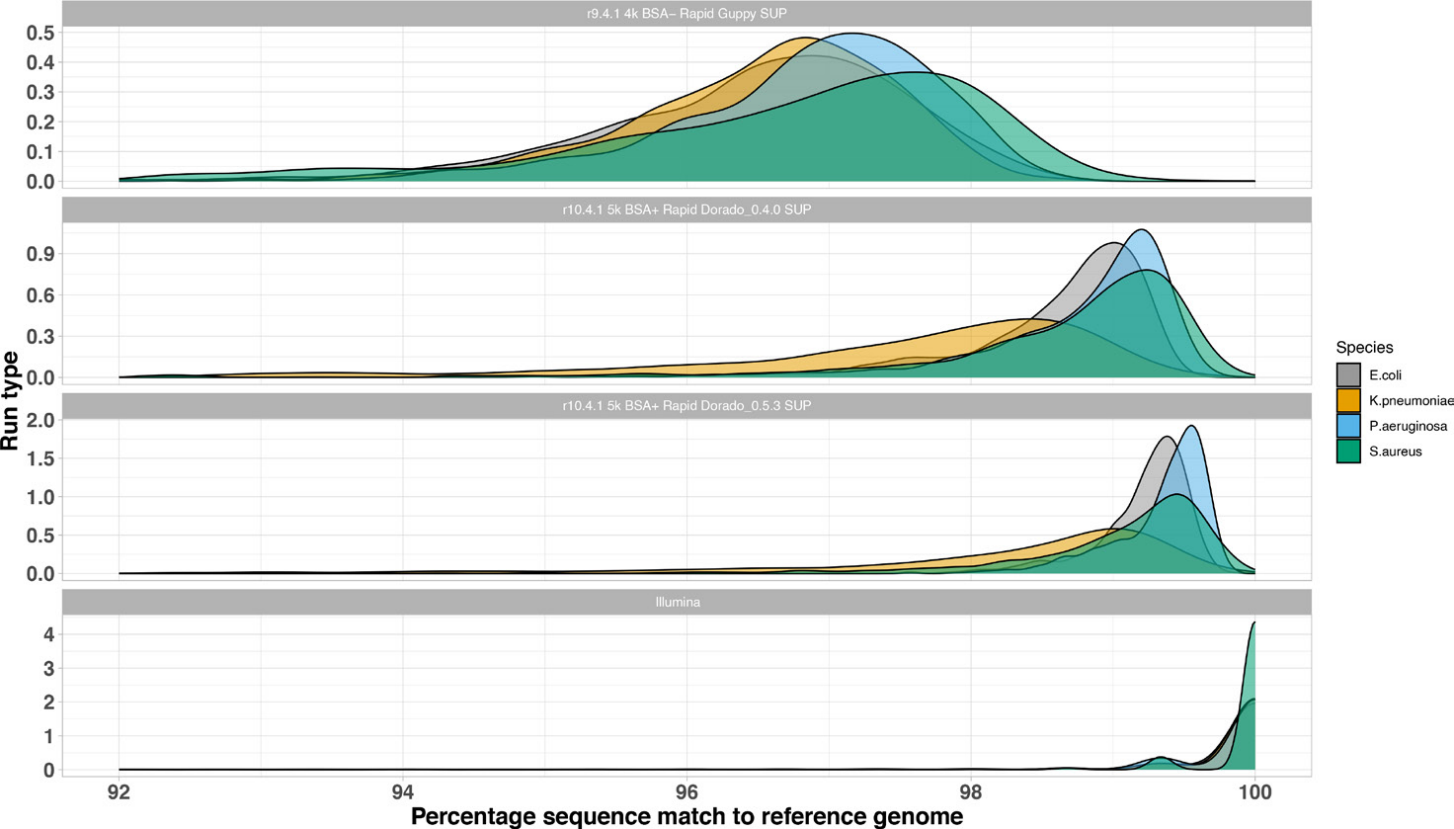
Distributions of read percentage match to the reference genome for four bacterial species reference strains by sequencing run type and bacterial species.

The empirical accuracy of the raw reads was determined from alignments to the hybrid Illumina/ONT Trycycler reference genomes (chromosomes only). Of the long-read methods, the R9.4.1 reads had the lowest accuracy (median: 96.8 % [IQR: 95.9–97.4 %]) compared with R10.4.1 reads (median: 98.8 %, [IQR: 98.1–99.2 %], *P*<0.001, two-sample Wilcoxon) 94 %; Fig. 1. The modal accuracies ranged from 97–97.5 % for R9.4.1 and increased to >99 % for the R10.4.1 reads. Reads basecalled using Dorado 0.5.3 showed higher read-level accuracies compared to the Dorado 0.4.0 data (median: 99.2 % [IQR: 98.8–99.5 %] vs median: 98.7 %, [IQR: 97.8–99.5 %], *P*<0.001, two-sample Wilcoxon).

### Four bacterial species reference strains: assembly chromosome and plasmid recovery

**Fig. 2.**
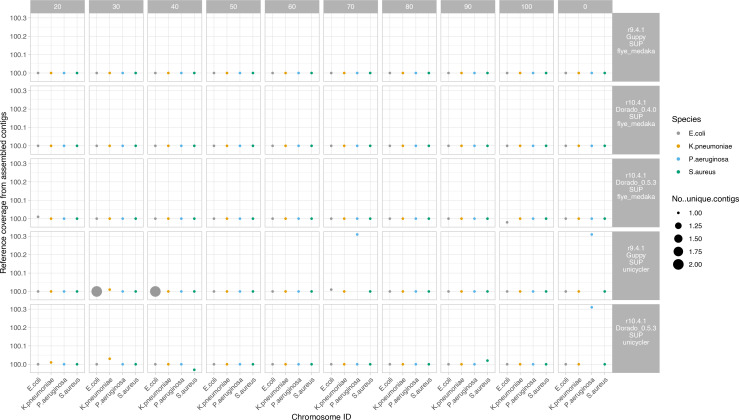
Percentage of reference strain chromosomes recovered by run type and species. Size of dots represent the number of unique contigs recovered. Faceted columns represent subsampling to a specified depth (x coverage), with 0 the full number of reads from the run used. Faceted rows represent run type, including the flowcell cell used (R9.4.1 or R10.4.1), the basecaller used (Guppy or dorado), and the assembly strategy used (Flye+Medaka or Unicyler) Unicycler assemblies represent Nanopore–Illumina hybrid assemblies.

The R10.4.1 nanopore-only (Flye and medaka) and R10.4.1+Illumina hybrid (Unicycler) approaches all fully assembled the reference chromosome sequences into single contigs at all subsampled sequencing depths, Fig. 2. The R9.4.1+Illumina hybrid (Unicycler) approach reconstructed the *E. coli* chromosome as two contigs (as opposed to a fully resolved, single contig) at 30x and 40× subsampled depths but completely resolved the reference strain chromosomes at all other depths (Fig. 2).

**Fig. 3.**
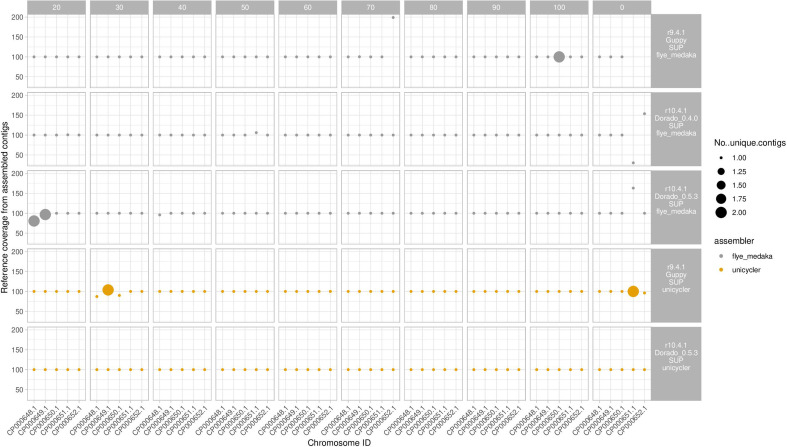
Percentage of reference strain *K. pneumoniae* plasmid sequences recovered by run type. Size of dots represent the number of unique contigs. Faceted columns represent subsampling to a specified depth (x coverage), with 0 the full number of reads from the run used. Faceted rows represent run type, including the flowcell cell used (R9.4.1 or R10.4.1), the basecaller used (Guppy or dorado), and the assembly strategy used (Flye+Medaka or Unicycler) Unicycler assemblies represent Nanopore–Illumina hybrid assemblies.

Plasmid recovery for most long-read-only assemblies was affected by either higher sequencing depth (i.e. impaired at >100×depth for R9.4.1 guppy/sup basecalled and R10.4.1 dorado_0.4.0 basecalled data) or at <30×sequencing depth (for R10.4.1 dorado_0.5.3 basecalled data), but was otherwise excellent (Fig. 3). Both R9.4.1+Illumina and R10.4.1+Illumina hybrid assemblies also resulted in excellent plasmid recovery with long-read sequencing depths of 20×–100×.

### Four bacterial species reference strains: assembly accuracy

**Fig. 4.**
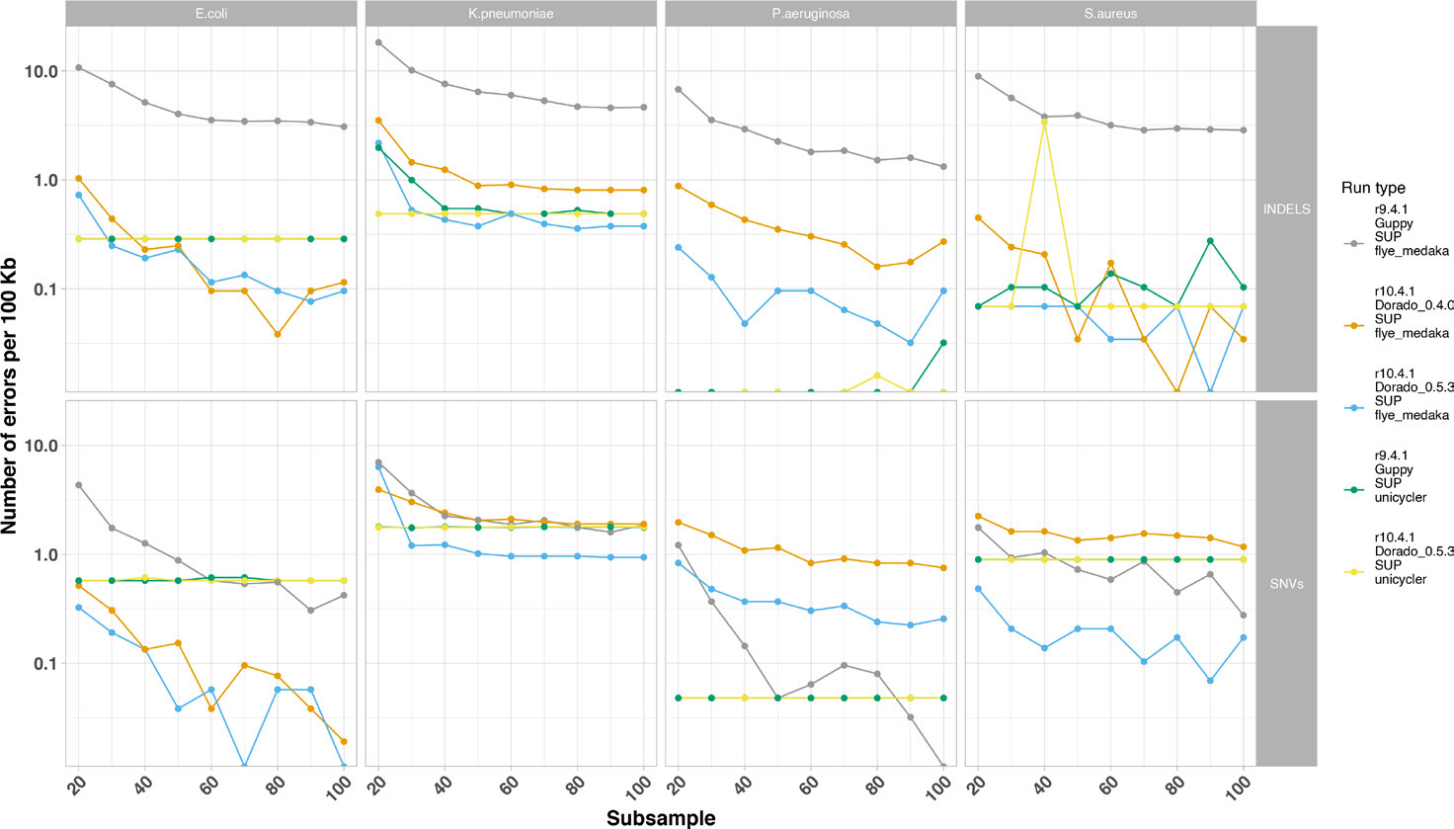
Number of errors by error type (i.e. faceted row: single nucleotide [SNV]; insertion or deletion [INDEL]). X-axis represents subsampling to a specified depth (x coverage). Colours represent run type, including the flowcell cell used (R9.4.1 or R10.4.1), the basecaller used (Guppy or dorado), and the assembly strategy used (Flye+Medaka or Unicycler). Unicycler assemblies represent Nanopore–Illumina hybrid assemblies.

Comparing the assembled contigs to the species reference genomes using dnadiff, the number of INDELs and SNV errors/100 kb was calculated (Figs 4 and S7; Table S1). Hybrid assemblies using R9.4.1+Illumina or R10.4.1+Illumina data had some of the lowest indel and SNV error rates, largely unaffected by long-read sequencing depth. Long-read-only assembly using R9.4.1 data (grey line, Fig. 4) had the highest number of indel errors ranging between 1 and >10 indels/100 kb, with some effect of increased sequencing depth in reducing this error rate. R10.4.1 long-read-only assemblies using data basecalled with the Dorado 0.5.3 (blue line, Fig. 4) consistently had the lowest indel and SNV error rates amongst long-read-only assemblies and for *E. coli, K. pneumoniae,* and *S. aureus,* these were lower than the hybrid assemblies.

**Fig. 5.**
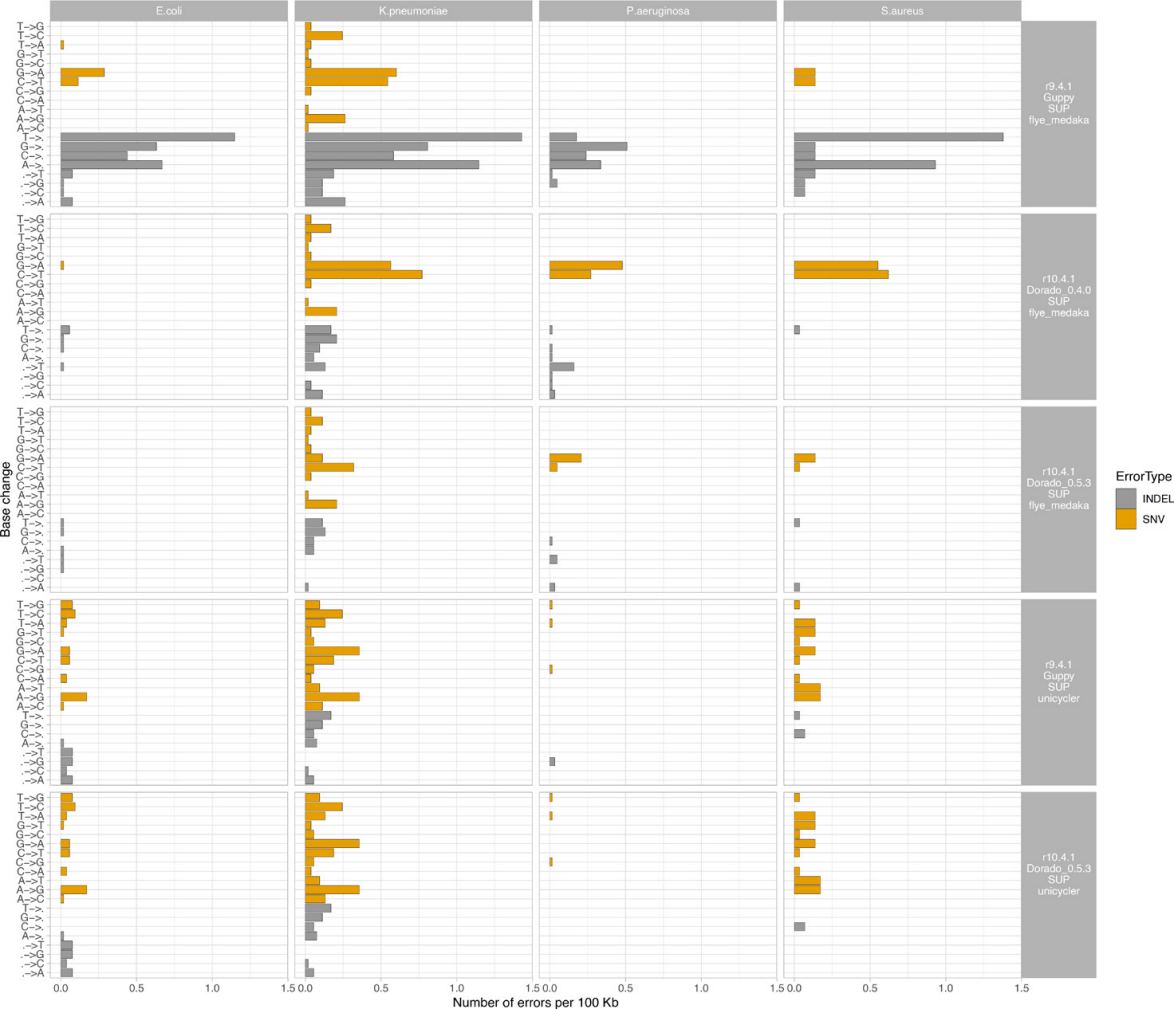
Number of SNV and indel errors per 100 Kb by base change or gain/loss event (changes from reference to assembly), by species (faceted columns). Faceted rows represent run type, including the flowcell cell used (R9.4.1 or R10.4.1), the basecaller used (Guppy or dorado), and the assembly strategy used (Flye+Medaka or Unicyler). Unicycler assemblies represent Nanopore–Illumina hybrid assemblies.

Indel rates were notably highest for R9.4.1 long-read only assemblies, and predominantly biassed to A and T deletions, except for *P. aeruginosa*, which notably has a high %GC content. These biases were less marked with R10.4.1 long-read-only assemblies, with much lower indel rates. At a SNV level, G-to-A and C-to-T SNV errors appeared systematically more frequent across most sequencing/basecalling/assembly approaches and for most species; for R10.4.1 long-read-only assemblies these were reduced by using the Dorado 0.5.3 basecaller (Fig. 5).

**Fig. 6.**
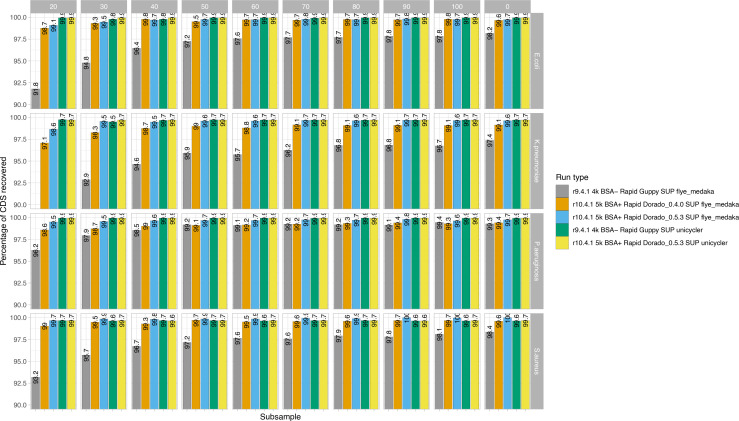
Bar chart showing the percentage of reference genome coding sequences (CDS) recovered by species (faceted rows) and subsampling to a specified depth (x coverage; faceted columns), with 0 the full number of reads from the run used. Colours represent run type, including the flowcell cell used (R9.4.1 or R10.4.1), the basecaller used (Guppy or dorado), and the assembly strategy used (Flye+Medaka or Unicycler). Unicycler assemblies represent Nanopore–Illumina hybrid assemblies.

Compared to the coding sequences (CDS) annotated in the hybrid trycycler-assembled reference genomes, CDS content recovery was excellent for long-read-only assemblies using R10.4.1 data and the Dorado 0.5.3 model (i.e. above 99 % at ≥30×coverage) and broadly comparable with gene content recovery for hybrid assemblies (Fig. 6).

### *E. coli* isolate sequencing

#### *E. coli* isolate sequencing: sequencing yield and read length distributions with and without BSA

The total sequencing yield for the run with BSA (BSA+) was 7.03 Gb, versus the run without BSA (BSA-) at 5.72 Gb. Following demultiplexing, ten isolates achieved enough bases for predicted ≥20×depth of coverage, however only nine empirically achieved this after mapping to the reference genomes and were used in further analysis (Fig. S8), although not all samples could be subsampled to 100×depth. Median (IQR) read lengths were shorter for the BSA +compared to BSA- groups: 2.61 Kb (IQR: 0.71–6.19 Kb) versus 3.42 Kb respectively (IQR: 1.28–7.12 Kb; *P*<0.001; two-sample Wilcoxon); (Figs S9 and S10). However, BSA treatment resulted in greater per-read accuracy across sequenced *E. coli* isolates (BSA +median %identity to the reference 99.32 % compared to BSA- median % identity 98.76 %; *P*<0.001, two sample Wilcoxon; Fig. S11.

#### *E. coli* isolate sequence assembly recovery and accuracy

**Fig. 7.**
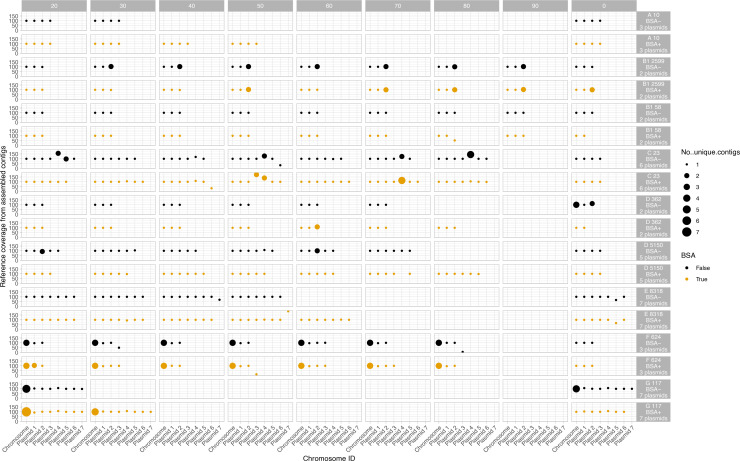
Percentage of reference chromosome and plasmids recovered for each of nine sequenced clinical *E. coli* isolates passing QC with (yellow) and without (black) BSA use in library treatment. Sequencing was done using R10.4.1+Dorado v0.5.3 basecalling and assembly with Flye and a single round of Medaka polishing (see Methods). Size of dots represent the number of unique contigs. Panels without any dots represent exclusions due to lack of coverage to subsample at this depth. The number of plasmids varies by isolate and plasmid number reflects the size order of plasmids within an isolate and does not correspond to the same plasmid between isolates. The expected number of plasmids based on the hybrid assemblies and Table 2, is denoted in the facet row label. The letter number combination preceding the isolate numeric identifier indicates the *E. coli* phylogroup of the isolate.

**Fig. 8.**
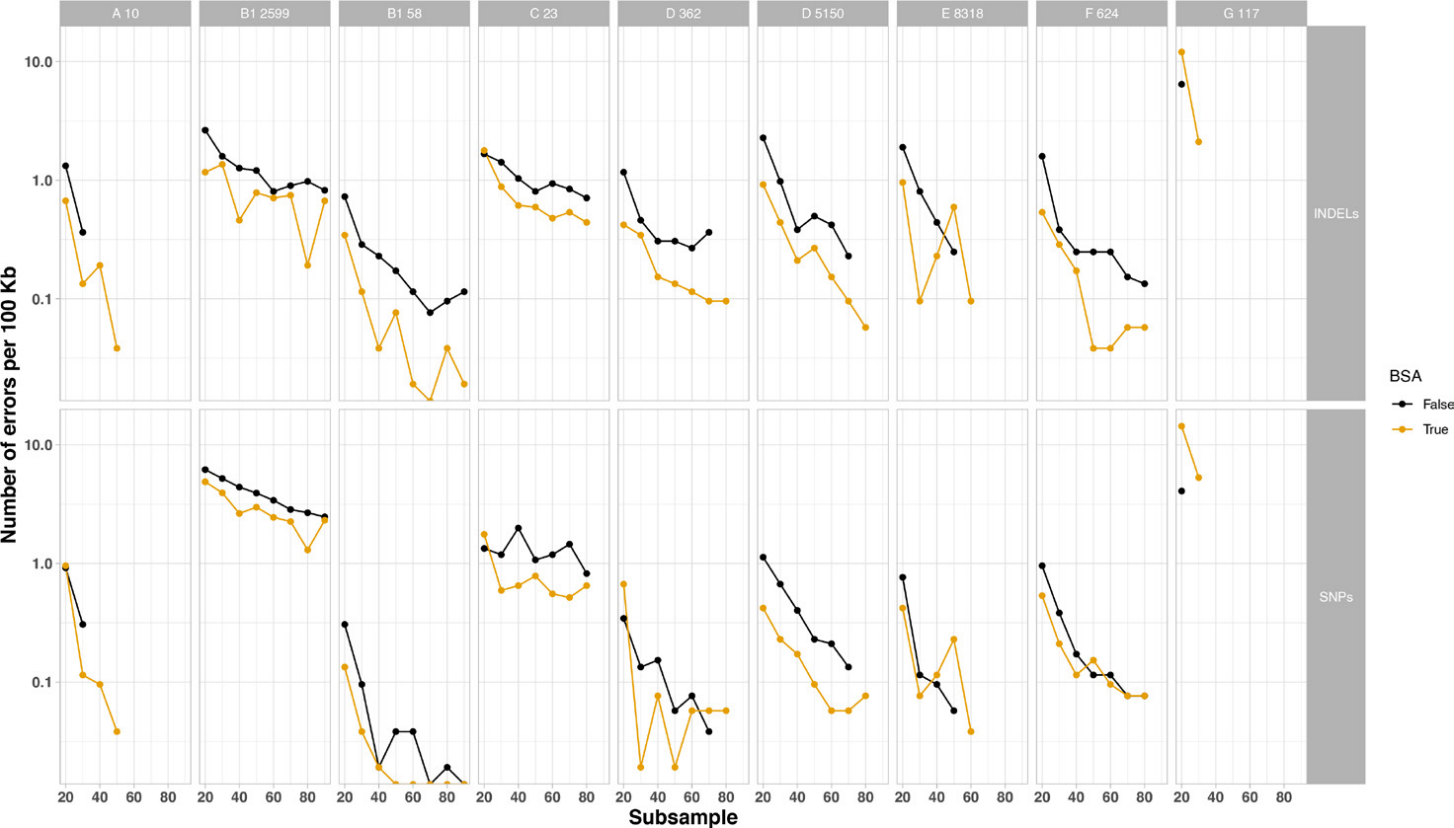
Number of errors per 100 kb over subsampled depth for each of nine sequenced clinical *E. coli* isolates passing QC with (yellow) and without (black) BSA use in library treatment. Rows show type, single nucleotide (SNV), insertion or deletion (INDEL). Faceted columns reflect different isolates. The letter number combination preceding the isolate numeric identifier indicates the *E. coli* phylogroup of the isolate. Absence of data represent insufficient data to subsample to this depth.

R9.4.1+Illumina hybrid reference assemblies for the nine *E. coli* isolates included in the final analysis had chromosome sizes ranging from 4.7 to 5.26 Mb and contained between 2–8 plasmids, ranging in size from ~1.5 Kb to ~150 Kb (Table 2). Subsequently, using R10.4.1 long-read-only assembly, overall chromosome and plasmid recovery was roughly equal irrespective of the use of BSA, where incomplete assemblies were generated for some isolates and did not improve with increased depth (Fig. 7). All chromosomes and plasmids were at least partially recovered at subsampling depths≥20×. However, BSA+ outputs showed a small reduction of indel and SNV error rates in 7/9 assembled genomes, particularly for indel error rates (Fig. 8).

**Table 2.** *E. coli* isolate reference chromosome and plasmid lengths (bp) from hybrid assemblies (R9.4.1+Illumina) for each of nine sequenced clinical *E. coli* isolates included in the analysis. The letter number combination preceding the isolate numeric identifier indicates the *E. coli* phylogroup of the isolate

Isolate name	Chromosome	Plasmid 1	Plasmid 2	Plasmid 3	Plasmid 4	Plasmid 5	Plasmid 6	Plasmid 7
**E 8318**	4 838 664	93 041	71 835	10 877	5 772	3 371	2 096	1 552
**F 624**	5 122 684	136 868	45 040	1 552				
**D 362**	5 094 307	137 038	4 671					
**A 10**	5 005 319	102 850	5 167	3 704				
**C 23**	4 701 962	145 252	99 455	46 065	2 328	2 101	1 545	
**B2 131**	5 007 009	83 691	69 217	6 082	5 167	4 087	1 822	1 565
**D 5150**	5 258 227	138 055	48 768	5 166	2 678	2 090		
**B1 58**	5 127 313	151 663	4 715					
**B1 2599**	4 715 961	134 924	98 497					
**G 117**	5 164 489	140 103	115 805	59 734	10 579	7 119	5 114	4 072

## Discussion

In this study we have shown that nanopore-only assemblies generated using R10.4.1 data to ~40× depth, basecalling with Dorado 0.5.3, and assembly with Flye and a single cycle of polishing with Medaka can be comparable to the high-throughput gold-standard approach of using Illumina-R9.4.1 or R10.4.1 nanopore hybrid assemblies (assembled using Unicycler). This reflects an iterative improvement over the R10.3 and R10.4 flowcells previously described (Fig. S12) [4], enabling accurate recovery of chromosomal and plasmid structures across the species evaluated.

We also observed that using BSA during the library preparation generally improved sequencing yields and read accuracy, representing potential savings for sequencing costs and laboratory time. In our hands the R10.4.1 flowcells (BSA+) generate ~6.4 Gb using the rapid library kit. The consumable cost of such a sequencing run is currently approximately £1200, resulting in a per isolate cost of £40–45 with 40×coverage Table 3.

**Table 3.** Approximate R10.4.1-only sequencing cost per isolate at varying sequencing depths and genome sizes. based on 5.9 Gb average yield from 3 R10.4.1 flowcells sequenced using the rapid library kit, and considering an approach using a 5 k sampling rate, Dorado basecalling and Flye assembly with one round of Medaka polishing

Desired coverage	Bp needed per genome	Max no. of genomes per flow cell	Cost per genome (£)
100x	500 000 000	11.8	101.14
90x	450 000 000	13.1	91.02
80x	400 000 000	14.7	80.91
70x	350 000 000	16.9	70.80
60x	300 000 000	19.7	60.68
50x	250 000 000	23.6	50.57
40x	200 000 000	29.5	40.45
30x	150 000 000	39.3	30.34
20x	100 000 000	59.0	20.23
10x	50 000 000	118.0	10.11

In our experience, R10 sequencing runs, including R10.3, R10.4 and R10.4.1, have consistently produced shorter read lengths than sequencing the same extracts with R9.4.1 flowcells/chemistries. DNA extracts have been generated from new cultures for the R10.4.1 sequencing run, therefore not affected by freeze-thawing fragmenting the DNA. However, batch effects from independently extracting the DNA for each run may have influenced the fragment sizes. To better understand this, multiple replicates would be needed for both R9.4.1 and R10.4.1 sequencing using the same DNA extracts.

### Limitations

There are several limitations with this study. Our results are based on outputs from single runs only and there are no replicates for each sample and flowcell type; however, our findings of species-specific differences in sequencing outputs are similar to those observed by others [6,20, 21]. Sequencing yield can be affected by numerous factors, including flowcell quality and baseline pore numbers, library preparation type, and operator skill. Replicating these findings by sequencing the same extracts using the same process across runs is important further work.

In this study we have compared annotated coding sequences between assembled genomes and the reference genome using a highly conservative requirement for a 100 % match; gene presence/absence studies may require less stringent thresholds. Similarly, different use cases may tolerate higher indel/SNV error rates – as a result we have presented these without applying arbitrary acceptability thresholds.

Although we observed species-specific differences in error rates and assembly quality, we have only investigated single isolates from three species and 13 *E. coli* isolates. Our findings may not be generalizable across other strains and species, and we would advise that users consider characterizing error rates and assembly quality for whichever species they are investigating by triangulating against a different method. Of note, there are regular updates to aspects of the ONT workflow such as basecallers and models, and we have not exhaustively investigated all possible combinations of sequencing, basecalling, assembly, and polishing strategy here, but have provided the raw data and reference assemblies to enable other users to evaluate their own workflows and updates.

In the context of these limitations however, we have found that R10.4.1/Kit 14 nanopore sequencing combined with updated, methylation-aware, basecalling models have improved bacterial genome reconstruction to enable reference quality genome assembly without the need for short-read sequencing and hybrid assembly. This could simplify pathogen genomics studies enabling cost-effective, higher-throughput and faster turnaround times.

## supplementary material

10.1099/mgen.0.001246 Uncited Supplementary Material 1.

## Abbreviations

bp: base pair
BSA: Bovine Serum Albumin
CDS: coding sequence
DNA: deoxyribonucleic acid
Gb: Giga base pairs
GC-content: guanine-cytosine content
INDEL: Insertion or deletion
IQR: Interquartile range
kb: kilo base pairs
kHz: Kilohertz
Mb: Mega base pairs
MRSA252: Methicillin-resistant Staphylococcus aureus strain 252
ONT: Oxford Nanopore Technologies
QC: Quality contro
SNP: Single nucleotide polymorphism
SNV: Single nucleotide variation
