# Supplementary material for: Evaluation of the accuracy of bacterial genome reconstruction with Oxford Nanopore R10.4.1 long-read-only sequencing
Source: Microb Genom. 2024 May 7;10(5):001246. doi: 10.1099/mgen.0.001246 (PMC11170131; doi:10.1099/mgen.0.001246)
Supplement: Uncited Supplementary Material 1. [file mgen-10-01246-s001.pdf]

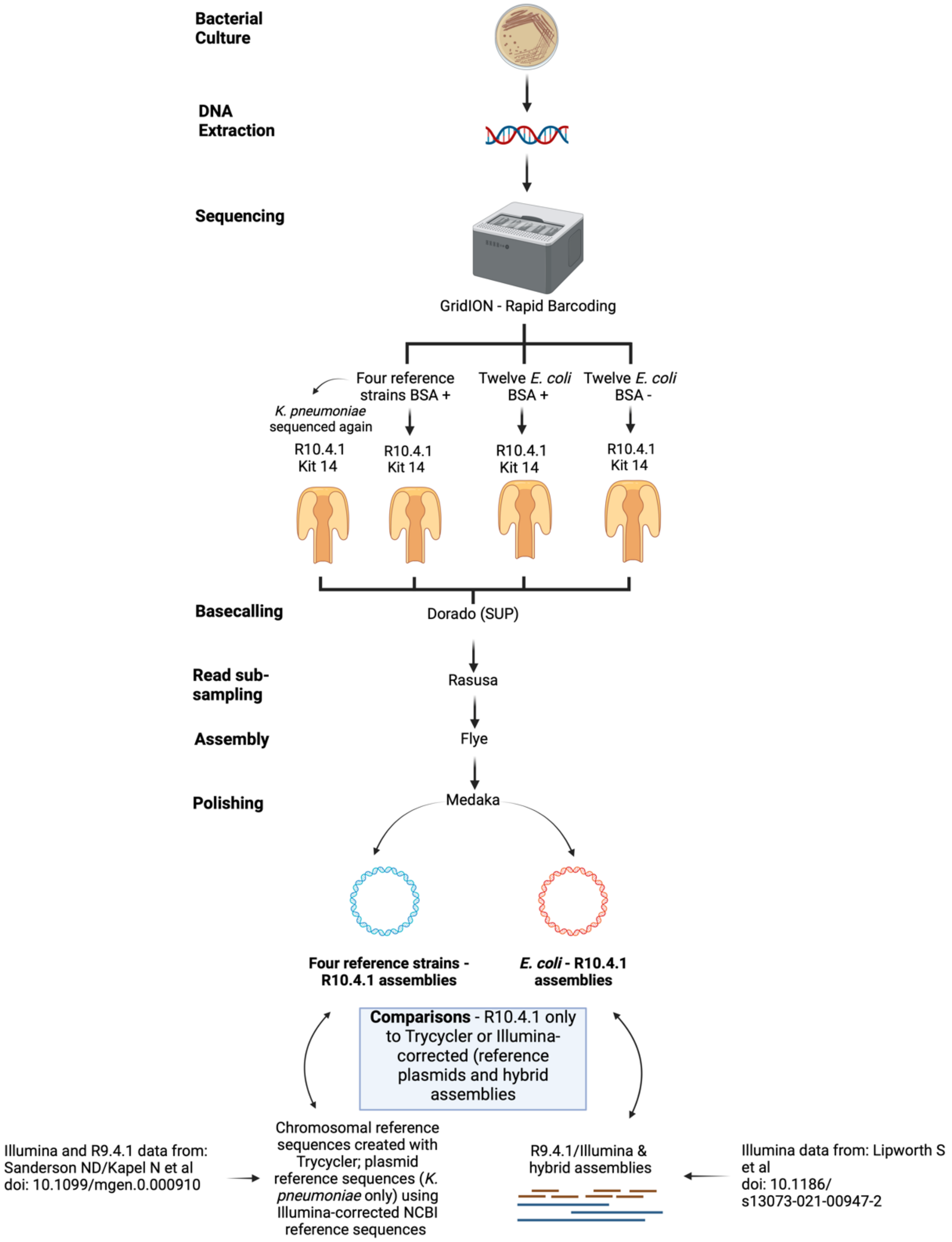

Supplementary figure S1. Schematic of experimental workflow.

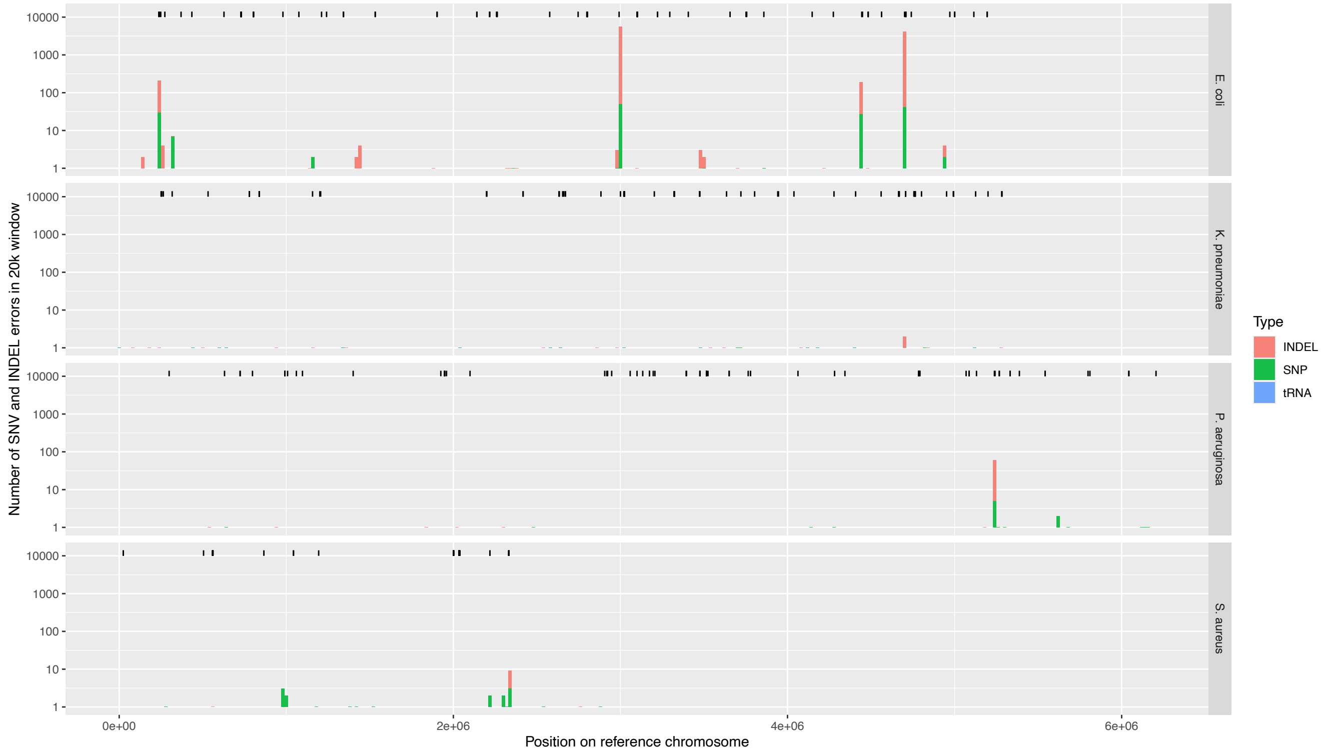

Supplementary figure S2. SNP and INDEL errors over the four Illumina corrected NCBI reference genomes compared against the best approach Flye-Medaka with 100x depth assemblies. Each position is a bin of 20 kb. Black columns above the plots represent tRNA positions.

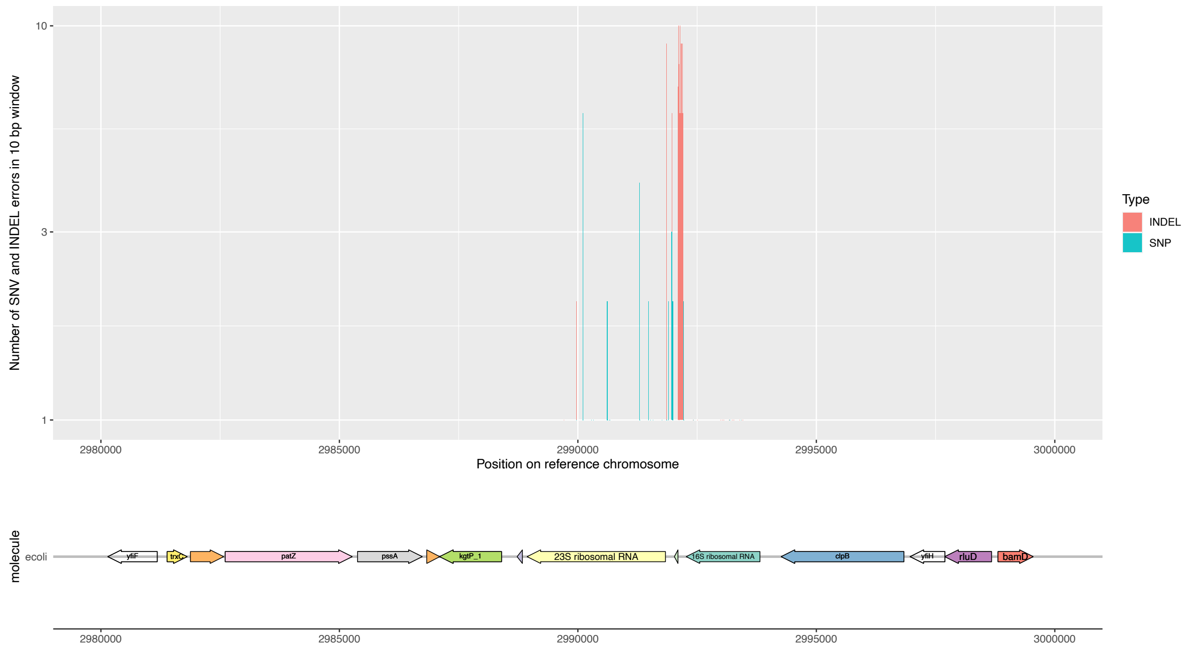

Supplementary figure S3. SNP and INDEL errors for the region 2.980 Mb to 3 Mb of *E. coli* genome shown in Supplementary figure S2. Each position represents a bin of 10 bases.

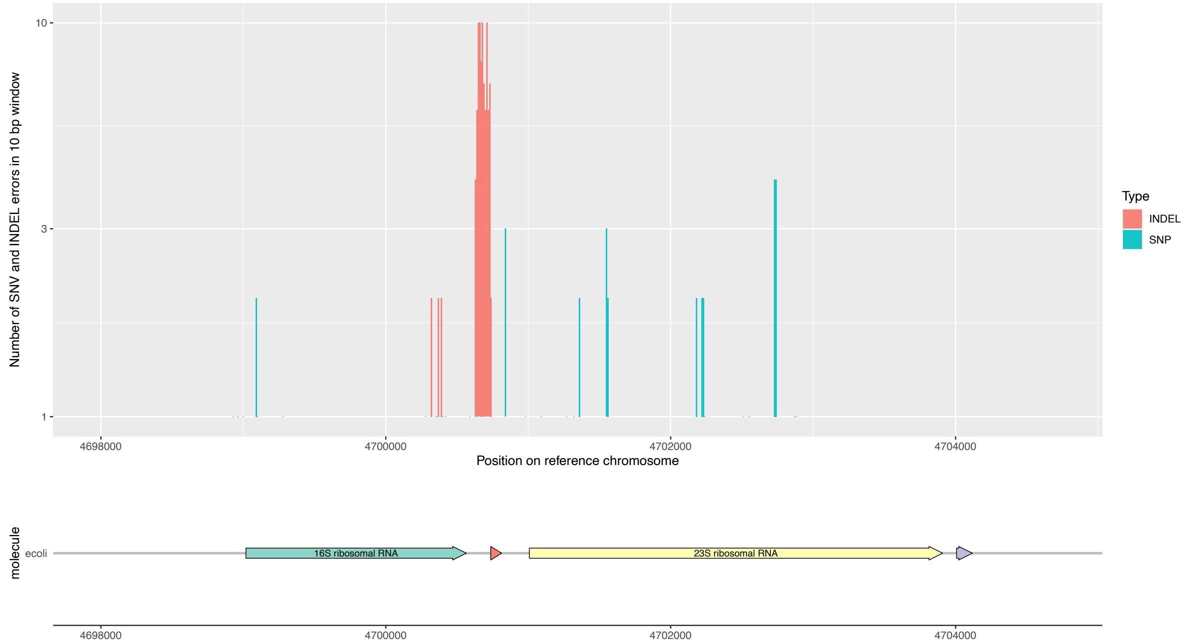

Supplementary figure S4. SNP and INDEL errors for the region 4.698 Mb to 4.704 Mb of *E. coli* genome shown in Supplementary figure S2. Each position represents a bin of 10 bases.

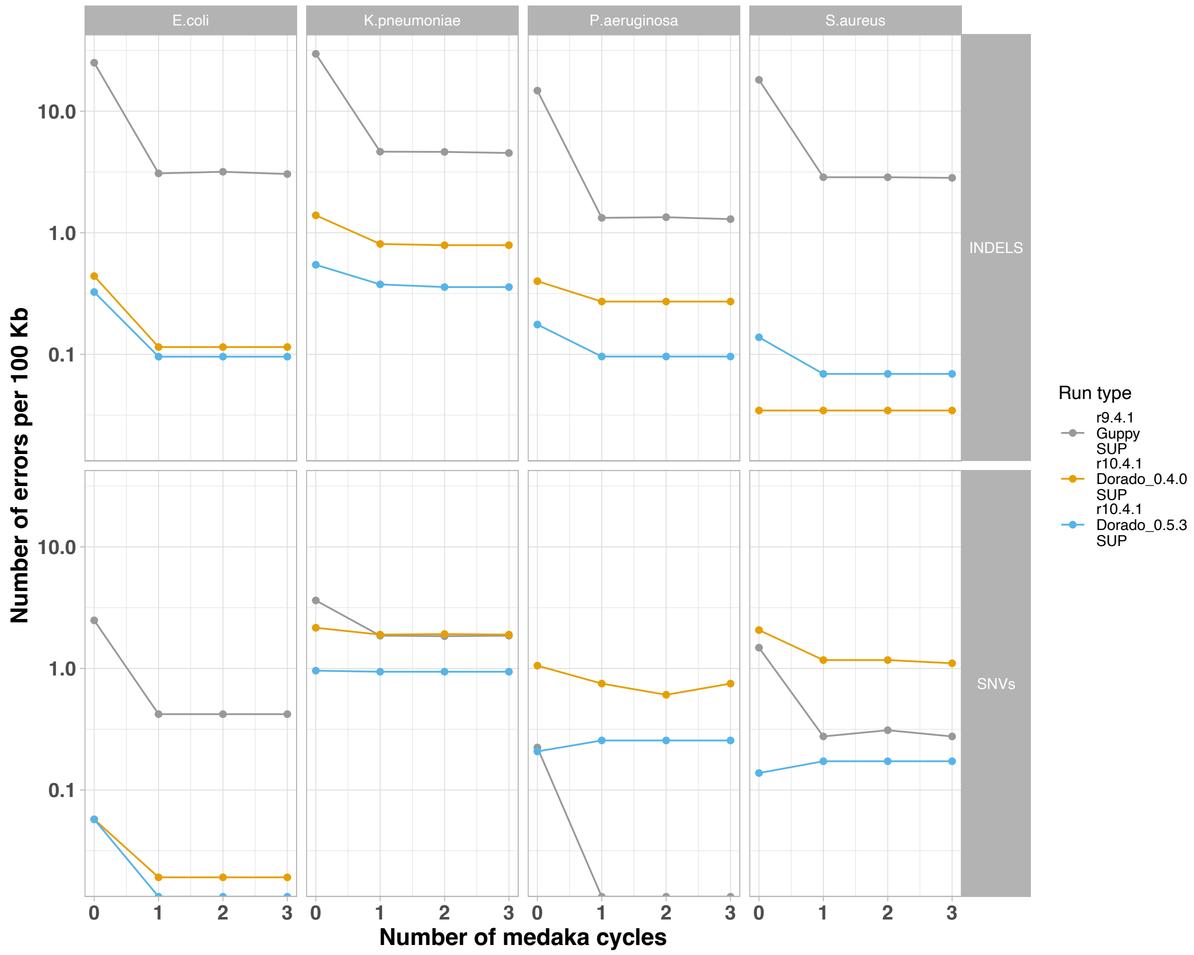

Supplementary figure S5. Number of errors per 100kb by species and sequencing/basecalling approach using 1, 2, or 3 cycles of Medaka polishing.

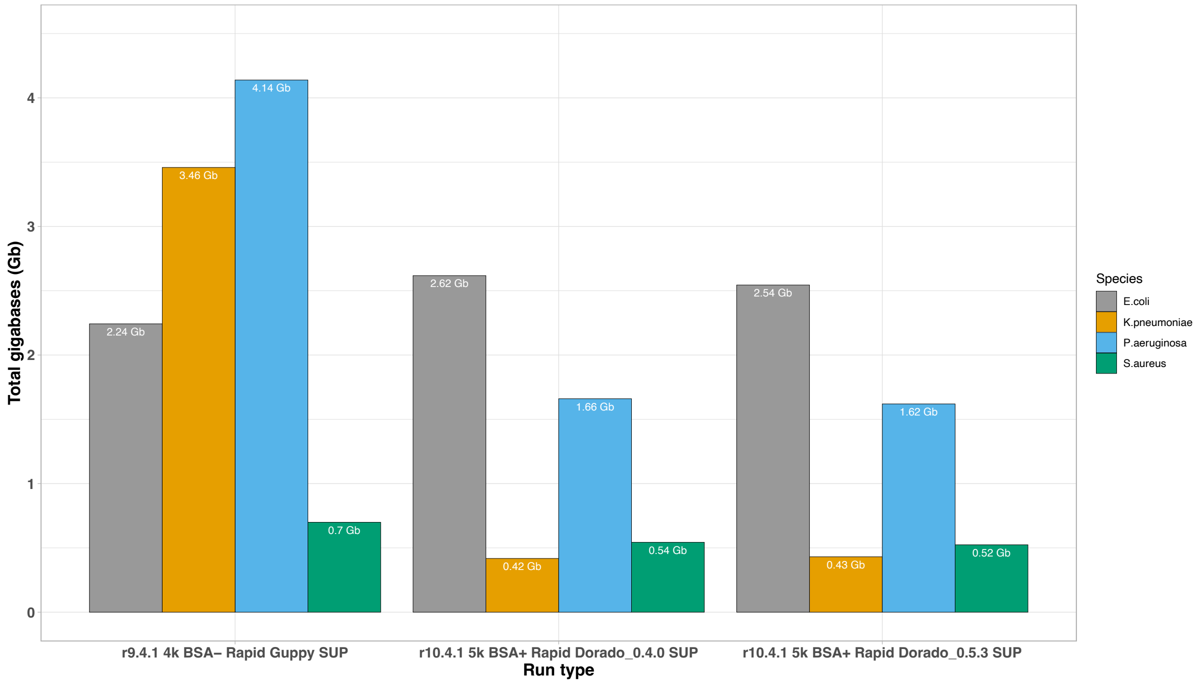

Supplementary figure S6. Bar plot showing sequencing yield for reference strains sequenced, for each species by run type, including the flowcell cell used (R9.4.1 or R10.4.1), sampling rate (4k or 5k), if BSA was used (BSA+ or BSA-), and the basecaller used (Guppy or dorado).

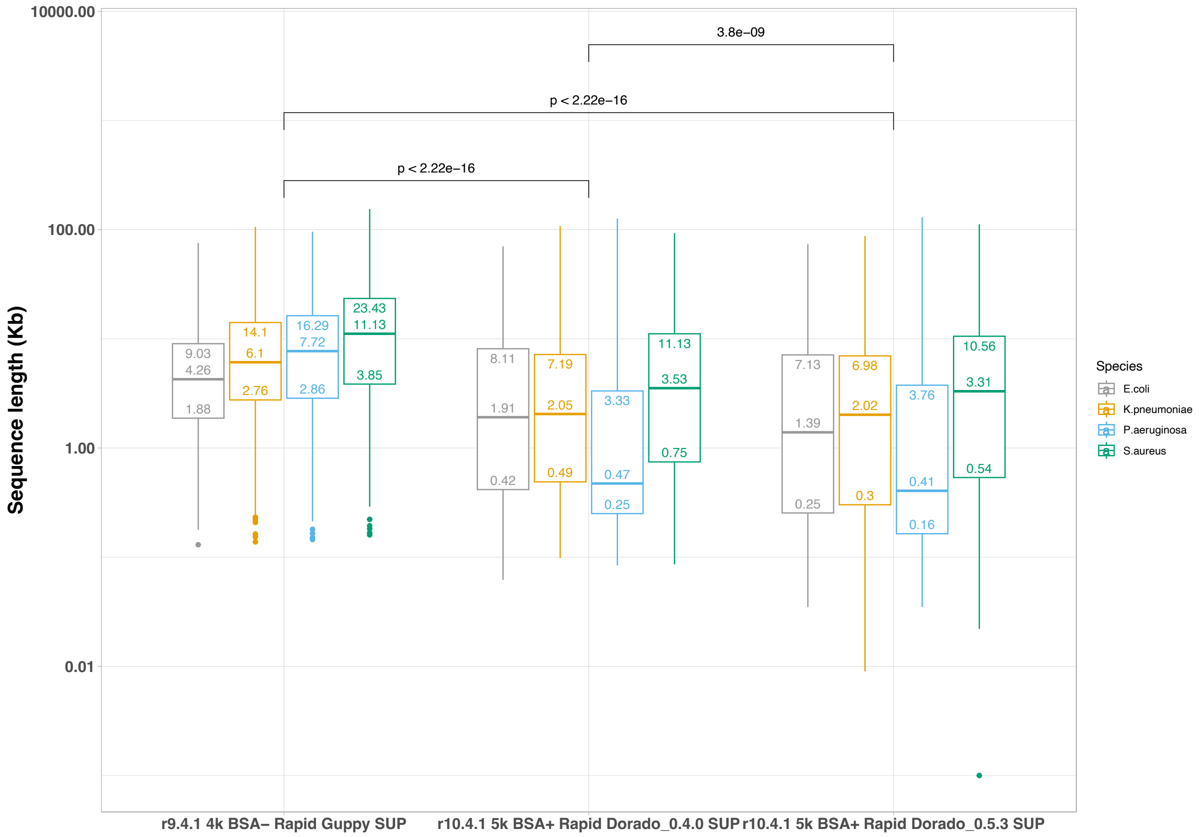

Supplementary figure S7. Box and whisker plot of sequencing read lengths for reference strains sequenced, by sequencing run type and bacterial species. P-values calculated using two-sample Wilcoxon test.

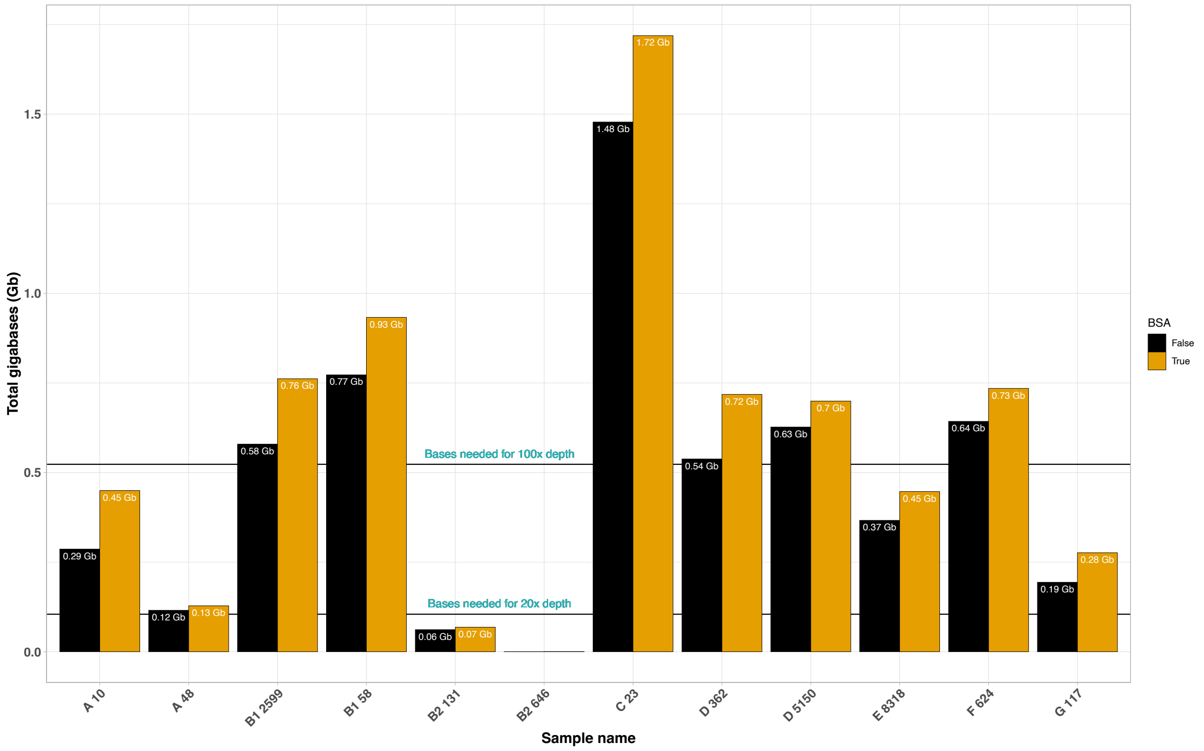

Supplementary figure S8. Number of bases generated for each of twelve sequenced clinical *E. coli* isolates, with bovine serum albumin (BSA) (yellow) and without BSA used as part of library preparation (black). Lines showing number of bases required for theoretical depths of 20x and 100x coverage. The letter number combination preceding the isolate numeric identifier indicates the *E. coli* phylogroup of the isolate.

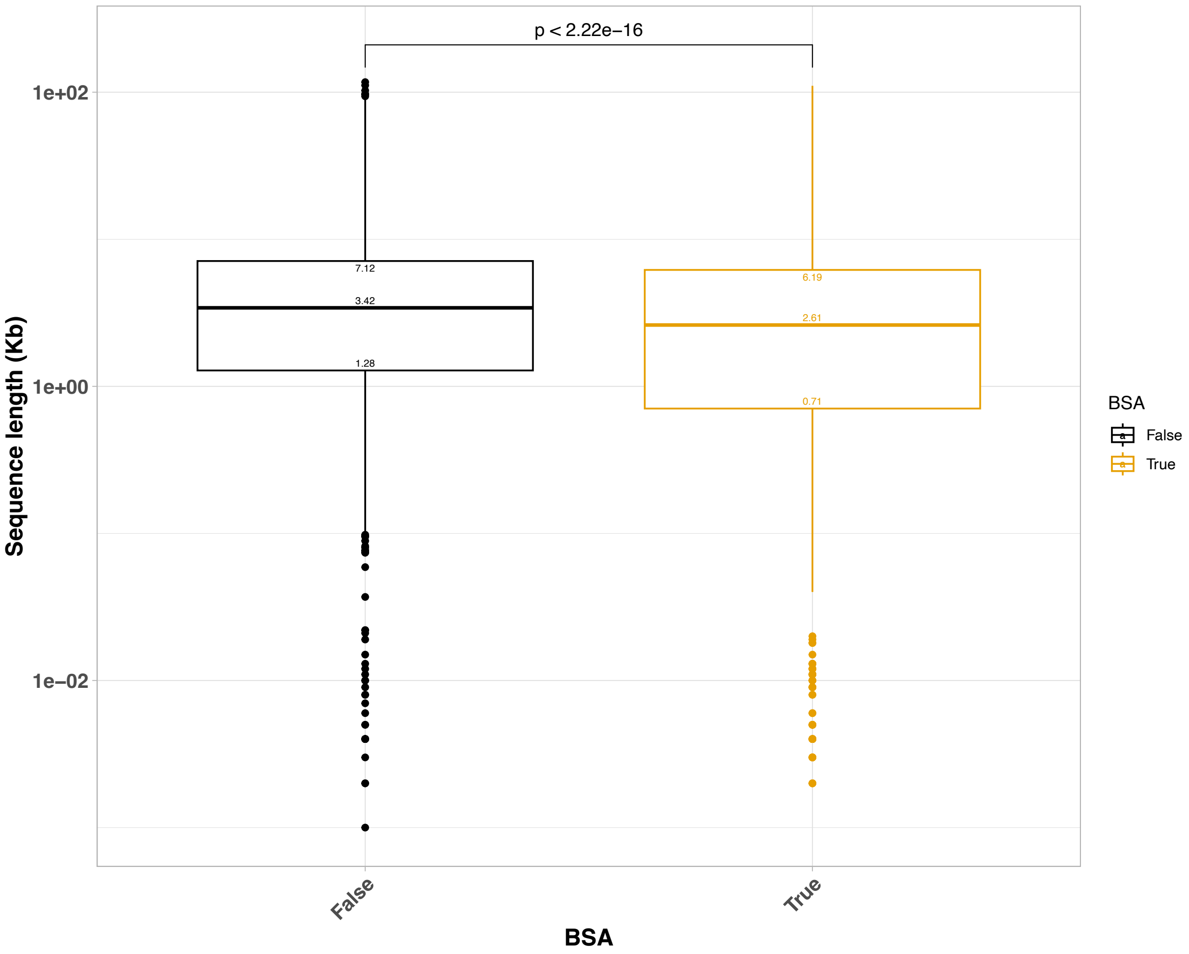

Supplementary figure S9. Box and whisker plot of read lengths of 9 successful *E. coli* isolates sequenced with or without BSA. P-value calculated with two sample Wilcoxon test.

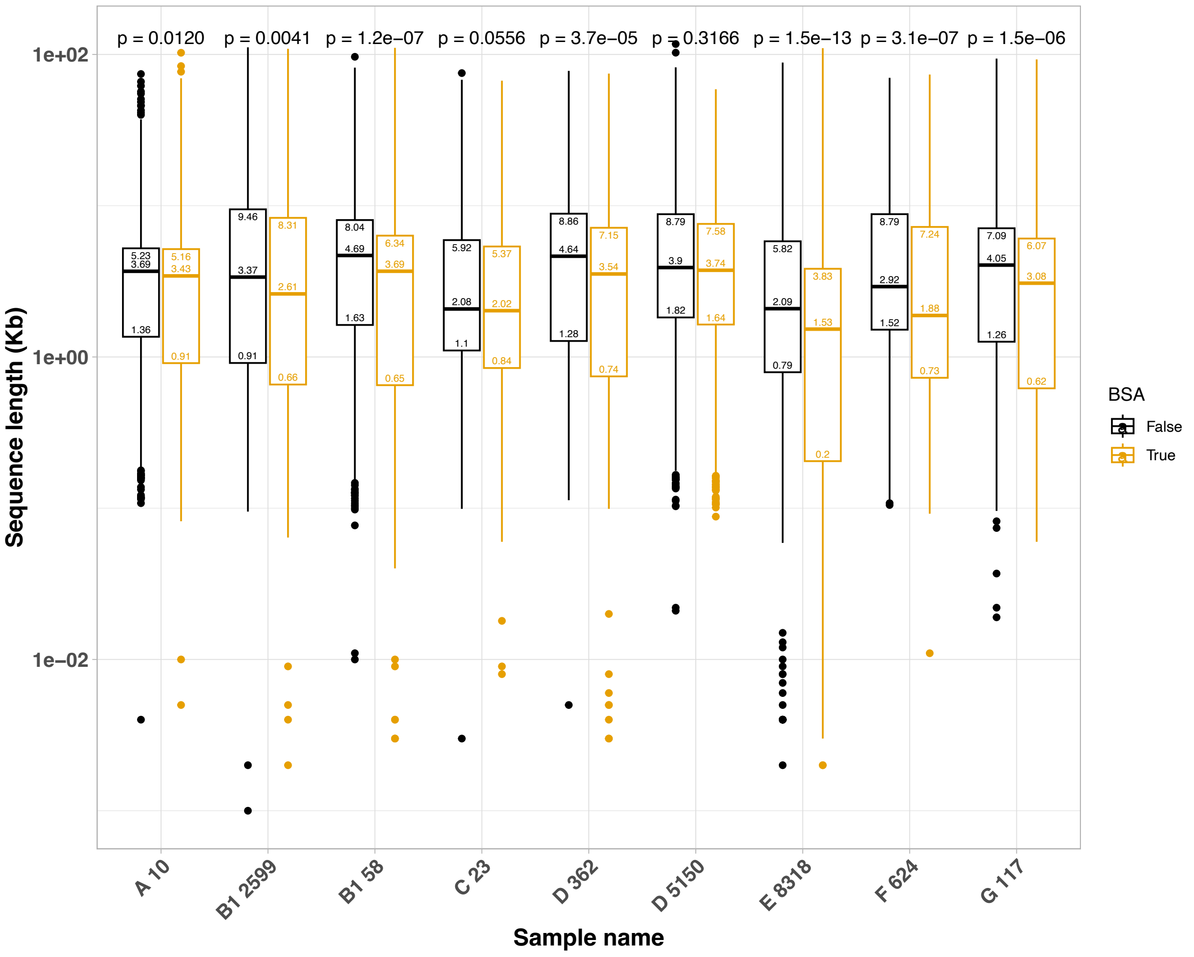

Supplementary figure S10. Read length box and whisker plots for each of nine sequenced clinical *E. coli* isolates passing QC with (yellow) and without (black) BSA use in library preparation. P value calculated using an unpaired two sample Wilcoxon test. The letter number combination preceding the isolate numeric identifier indicates the *E. coli* phylogroup of the isolate.

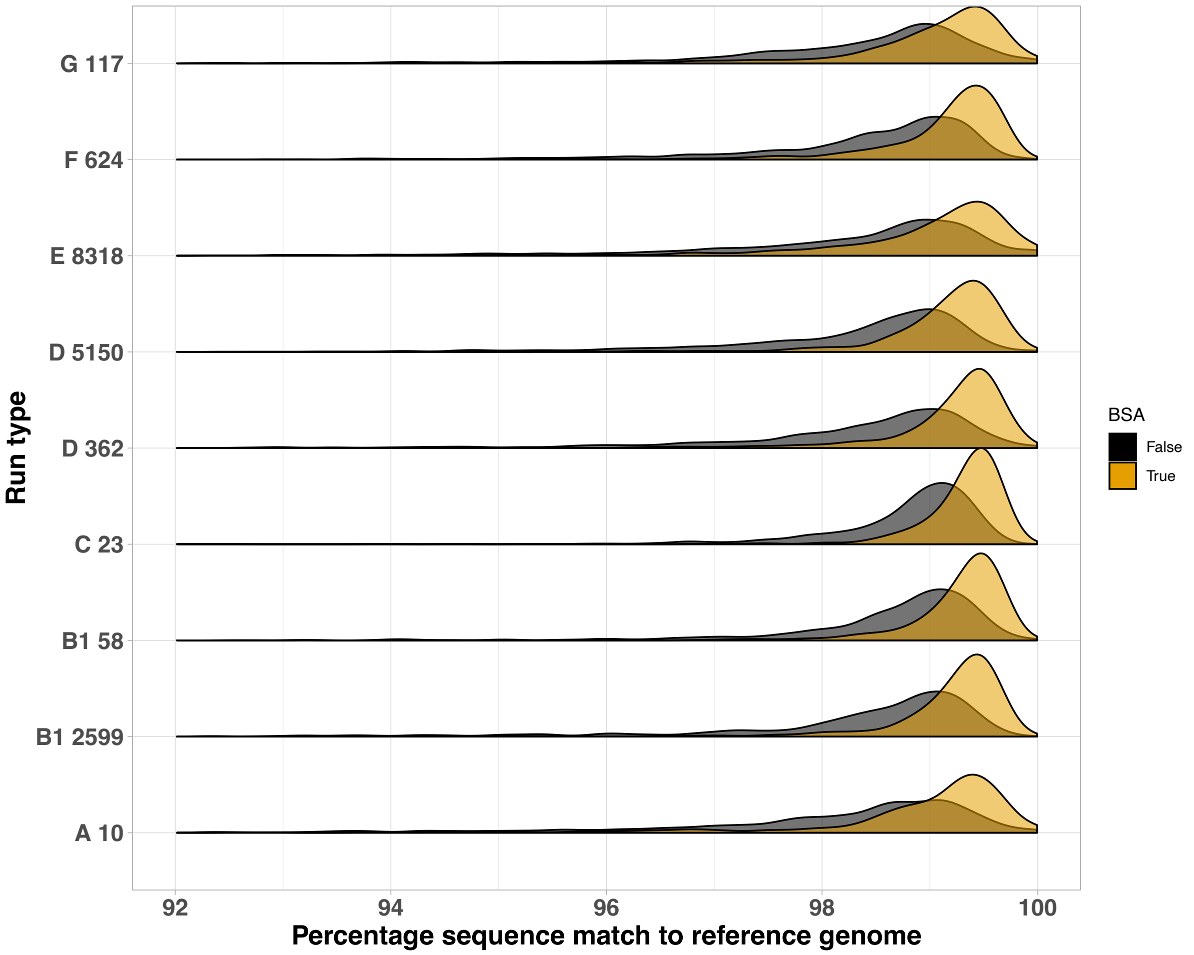

Supplementary figure S11. Distributions of percentage read match to the reference genomes for each of nine sequenced clinical *E. coli* isolates passing QC with (yellow) and without (black) BSA use in library preparation. The letter number combination preceding the isolate numeric identifier indicates the *E. coli* phylogroup of the isolate.

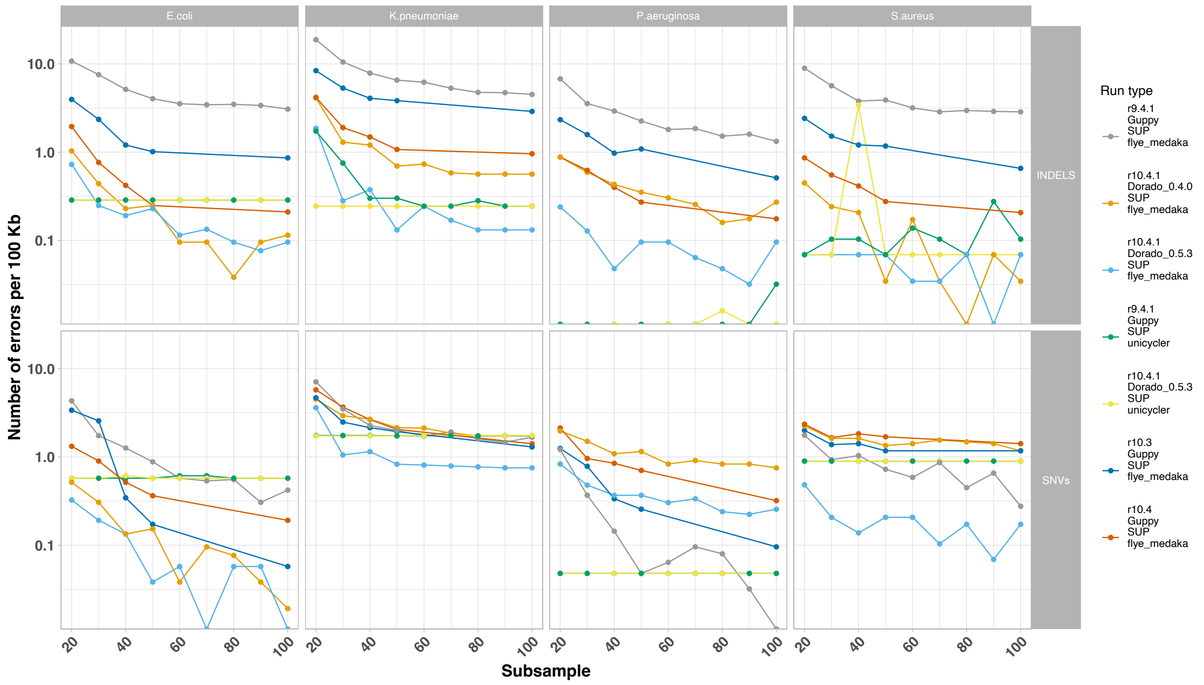

Supplementary figure S12. Number of errors by error type (i.e. faceted row: single nucleotide [SNV]; insertion or deletion [INDEL]). X-axis represents subsampling to a specified depth (x coverage). Colours represent run type, including the flowcell cell used (R9.4.1, R10.3, R10.4, or R10.4.1), the basecaller used (Guppy or dorado), and the assembly strategy used (Flye+Medaka or Unicycler). Unicycler assemblies represent nanopore-Illumina hybrid assemblies.

Run type	subsample	type	q1	median	q3
r10.4.1 Dorado_0.4.0 SUP flye_medaka	20	INDELs	0.77101465	0.95554951	1.65313798
r10.4.1 Dorado_0.4.0 SUP flye_medaka	20	SNPs	1.60270527	2.10311777	2.66317734
r10.4.1 Dorado_0.4.0 SUP flye_medaka	30	INDELs	0.39017274	0.51541243	0.80513284
r10.4.1 Dorado_0.4.0 SUP flye_medaka	30	SNPs	1.20267992	1.56114355	1.97209619
r10.4.1 Dorado_0.4.0 SUP flye_medaka	40	INDELs	0.22380827	0.33037771	0.6336328
r10.4.1 Dorado_0.4.0 SUP flye_medaka	40	SNPs	0.84815729	1.35347582	1.817021
r10.4.1 Dorado_0.4.0 SUP flye_medaka	50	INDELs	0.19504516	0.30000183	0.48444294
r10.4.1 Dorado_0.4.0 SUP flye_medaka	50	SNPs	0.90086073	1.24749367	1.52083873
r10.4.1 Dorado_0.4.0 SUP flye_medaka	60	INDELs	0.15321092	0.23796409	0.45319969
r10.4.1 Dorado_0.4.0 SUP flye_medaka	60	SNPs	0.63256342	1.12223201	1.58666062
r10.4.1 Dorado_0.4.0 SUP flye_medaka	70	INDELs	0.08032241	0.17559667	0.39846021
r10.4.1 Dorado_0.4.0 SUP flye_medaka	70	SNPs	0.70680792	1.23113363	1.65721416
r10.4.1 Dorado_0.4.0 SUP flye_medaka	80	INDELs	0.02868069	0.09899266	0.32187598
r10.4.1 Dorado_0.4.0 SUP flye_medaka	80	SNPs	0.64212365	1.15671477	1.58669303
r10.4.1 Dorado_0.4.0 SUP flye_medaka	90	INDELs	0.0889431	0.13566057	0.33385681
r10.4.1 Dorado_0.4.0 SUP flye_medaka	90	SNPs	0.63256342	1.12223201	1.53496889
r10.4.1 Dorado_0.4.0 SUP flye_medaka	100	INDELs	0.09466275	0.19314412	0.40574179
r10.4.1 Dorado_0.4.0 SUP flye_medaka	100	SNPs	0.56787916	0.96160626	1.3539344

r10.4.1 Dorado_0.5.3 SUP flye_medaka	20	INDELs	0.19695384	0.48309703	1.09004586
r10.4.1 Dorado_0.5.3 SUP flye_medaka	20	SNPs	0.44333092	0.65671477	2.20664981
r10.4.1 Dorado_0.5.3 SUP flye_medaka	30	INDELs	0.11308802	0.18818075	0.31800342
r10.4.1 Dorado_0.5.3 SUP flye_medaka	30	SNPs	0.20297356	0.34306489	0.6601768
r10.4.1 Dorado_0.5.3 SUP flye_medaka	40	INDELs	0.06370497	0.13008505	0.25148615
r10.4.1 Dorado_0.5.3 SUP flye_medaka	40	SNPs	0.13690908	0.25267159	0.58101023
r10.4.1 Dorado_0.5.3 SUP flye_medaka	50	INDELs	0.08912636	0.16264608	0.26606909
r10.4.1 Dorado_0.5.3 SUP flye_medaka	50	SNPs	0.16473264	0.28715435	0.5293185
r10.4.1 Dorado_0.5.3 SUP flye_medaka	60	INDELs	0.08050567	0.1052847	0.20822252
r10.4.1 Dorado_0.5.3 SUP flye_medaka	60	SNPs	0.16951276	0.25520546	0.46729744
r10.4.1 Dorado_0.5.3 SUP flye_medaka	70	INDELs	0.05654401	0.09887049	0.19906662
r10.4.1 Dorado_0.5.3 SUP flye_medaka	70	SNPs	0.07758621	0.21945577	0.4912591
r10.4.1 Dorado_0.5.3 SUP flye_medaka	80	INDELs	0.06370497	0.08228391	0.16098744
r10.4.1 Dorado_0.5.3 SUP flye_medaka	80	SNPs	0.14365069	0.2060152	0.41937411
r10.4.1 Dorado_0.5.3 SUP flye_medaka	90	INDELs	0.02396166	0.05421536	0.15134634
r10.4.1 Dorado_0.5.3 SUP flye_medaka	90	SNPs	0.06606448	0.14630384	0.40269404
r10.4.1 Dorado_0.5.3 SUP flye_medaka	100	INDELs	0.0889431	0.09572447	0.16586995
r10.4.1 Dorado_0.5.3 SUP flye_medaka	100	SNPs	0.12931034	0.21400242	0.4266557
r10.4.1 Dorado_0.5.3 SUP unicycler	20	INDELs	0.05172414	0.1778862	0.33728561
r10.4.1 Dorado_0.5.3 SUP unicycler	20	SNPs	0.44219116	0.73508275	1.11414312
r10.4.1 Dorado_0.5.3 SUP unicycler	30	INDELs	0.05172414	0.1778862	0.33728561
r10.4.1 Dorado_0.5.3 SUP unicycler	30	SNPs	0.44219116	0.73508275	1.11414312
r10.4.1 Dorado_0.5.3 SUP unicycler	40	INDELs	0.21510516	0.38776434	1.21998963
r10.4.1 Dorado_0.5.3 SUP unicycler	40	SNPs	0.47087184	0.7542032	1.11414312
r10.4.1 Dorado_0.5.3 SUP unicycler	50	INDELs	0.05172414	0.1778862	0.33728561
r10.4.1 Dorado_0.5.3 SUP unicycler	50	SNPs	0.44219116	0.73508275	1.11414312
r10.4.1 Dorado_0.5.3 SUP unicycler	60	INDELs	0.05172414	0.1778862	0.33728561
r10.4.1 Dorado_0.5.3 SUP unicycler	60	SNPs	0.44219116	0.73508275	1.11884236
r10.4.1 Dorado_0.5.3 SUP unicycler	70	INDELs	0.05172414	0.1778862	0.33728561
r10.4.1 Dorado_0.5.3 SUP unicycler	70	SNPs	0.44219116	0.73508275	1.11884236
r10.4.1 Dorado_0.5.3 SUP unicycler	80	INDELs	0.05571775	0.1778862	0.33728561
r10.4.1 Dorado_0.5.3 SUP unicycler	80	SNPs	0.44219116	0.73508275	1.11884236
r10.4.1 Dorado_0.5.3 SUP unicycler	90	INDELs	0.05172414	0.1778862	0.33728561
r10.4.1 Dorado_0.5.3 SUP unicycler	90	SNPs	0.44219116	0.73508275	1.11884236
r10.4.1 Dorado_0.5.3 SUP unicycler	100	INDELs	0.05172414	0.1778862	0.33728561
r10.4.1 Dorado_0.5.3 SUP unicycler	100	SNPs	0.44219116	0.73508275	1.11884236
r9.4.1 Guppy SUP flye_medaka	20	INDELs	8.3915666	9.83836619	12.6222433
r9.4.1 Guppy SUP flye_medaka	20	SNPs	1.62247989	3.0399222	4.98903808
r9.4.1 Guppy SUP flye_medaka	30	INDELs	5.12796078	6.60387684	8.20202993
r9.4.1 Guppy SUP flye_medaka	30	SNPs	0.7901289	1.33549812	2.21662545
r9.4.1 Guppy SUP flye_medaka	40	INDELs	3.57565826	4.46825344	5.75604882
r9.4.1 Guppy SUP flye_medaka	40	SNPs	0.81180456	1.14821652	1.51037249

r9.4.1 Guppy SUP flye_medaka	50	INDELs	3.48551283	3.96548428	4.63295548
r9.4.1 Guppy SUP flye_medaka	50	SNPs	0.55508428	0.80183952	1.17657312
r9.4.1 Guppy SUP flye_medaka	60	INDELs	2.8305883	3.35484934	4.15202382
r9.4.1 Guppy SUP flye_medaka	60	SNPs	0.44618477	0.57991033	0.90957998
r9.4.1 Guppy SUP flye_medaka	70	INDELs	2.60981051	3.15187578	3.91114917
r9.4.1 Guppy SUP flye_medaka	70	SNPs	0.4254913	0.69872091	1.15876977
r9.4.1 Guppy SUP flye_medaka	80	INDELs	2.6035309	3.22272038	3.78475467
r9.4.1 Guppy SUP flye_medaka	80	SNPs	0.35617495	0.50138458	0.8575993
r9.4.1 Guppy SUP flye_medaka	90	INDELs	2.57177482	3.14043647	3.68485746
r9.4.1 Guppy SUP flye_medaka	90	SNPs	0.23743273	0.48054988	0.8908154
r9.4.1 Guppy SUP flye_medaka	100	INDELs	2.47802137	2.97023142	3.4695097
r9.4.1 Guppy SUP flye_medaka	100	SNPs	0.20689655	0.34825608	0.78071314
r9.4.1 Guppy SUP unicycler	20	INDELs	0.05172414	0.1778862	0.70852622
r9.4.1 Guppy SUP unicycler	20	SNPs	0.44219116	0.73508275	1.11884236
r9.4.1 Guppy SUP unicycler	30	INDELs	0.07758621	0.19512758	0.46416531
r9.4.1 Guppy SUP unicycler	30	SNPs	0.44219116	0.73508275	1.10944387
r9.4.1 Guppy SUP unicycler	40	INDELs	0.07758621	0.19512758	0.35138336
r9.4.1 Guppy SUP unicycler	40	SNPs	0.44219116	0.73508275	1.11884236
r9.4.1 Guppy SUP unicycler	50	INDELs	0.05172414	0.1778862	0.35138336
r9.4.1 Guppy SUP unicycler	50	SNPs	0.44219116	0.73508275	1.11414312
r9.4.1 Guppy SUP unicycler	60	INDELs	0.10344828	0.21236896	0.33728561
r9.4.1 Guppy SUP unicycler	60	SNPs	0.47087184	0.7542032	1.11414312
r9.4.1 Guppy SUP unicycler	70	INDELs	0.07758621	0.19512758	0.33728561
r9.4.1 Guppy SUP unicycler	70	SNPs	0.47087184	0.7542032	1.11884236
r9.4.1 Guppy SUP unicycler	80	INDELs	0.05172414	0.1778862	0.34668411
r9.4.1 Guppy SUP unicycler	80	SNPs	0.44219116	0.73508275	1.11414312
r9.4.1 Guppy SUP unicycler	90	INDELs	0.20689655	0.28133448	0.33728561
r9.4.1 Guppy SUP unicycler	90	SNPs	0.44219116	0.73508275	1.11884236
r9.4.1 Guppy SUP unicycler	100	INDELs	0.08557343	0.19512758	0.33728561
r9.4.1 Guppy SUP unicycler	100	SNPs	0.44219116	0.73508275	1.11414312

Supplementary table S1. Errors per 100 Kb for each flow cell run types at different subsampling depths.
